# A multicenter phase II trial of primary prophylactic PEG‐rhG‐CSF in pediatric patients with solid tumors and non‐Hodgkin lymphoma after chemotherapy: An interim analysis

**DOI:** 10.1002/cam4.6079

**Published:** 2023-05-15

**Authors:** Junting Huang, Suying Lu, Juan Wang, Lian Jiang, Xuequn Luo, Xiangling He, Yanpeng Wu, Yi Wang, Xiuli Zhu, Jian Chen, Yanlai Tang, Keke Chen, Xin Tian, Boyun Shi, Lanying Guo, Jia Zhu, Feifei Sun, Zijun Zhen, Yizhuo Zhang

**Affiliations:** ^1^ Department of Pediatric Oncology, State Key Laboratory of Oncology in South China, Collaborative Innovation Center for Cancer Medicine Sun Yat‐Sen University Cancer Center Guangzhou P. R. China; ^2^ Department of Pediatrics Hebei Tumor Hospital, Pediatric Shijiazhuang P. R. China; ^3^ Department of Pediatrics The First Affiliated Hospital of Sun Yat‐sen University Guangzhou P. R. China; ^4^ Department of Pediatric Hematology and Oncology Hunan Provincial People's Hospital Changsha P. R. China; ^5^ Department of Pediatrics The Fifth Affiliated Hospital, Guangzhou Medical University Guangzhou P. R. China

**Keywords:** chemotherapy, febrile neutropenia, lymphoma, pediatric cancer, pegylated granulocyte colony‐stimulating factor

## Abstract

**Background:**

Pegylated recombinant human granulocyte colony‐stimulating factor (PEG‐rhG‐CSF) can be used in pediatric patients. This study assessed the safety and efficacy of PEG‐rhG‐CSF as a primary prophylactic drug against neutropenia after chemotherapy in pediatric patients with solid tumors or non‐Hodgkin lymphoma (NHL).

**Patients and Methods:**

This phase II study (between October 2020 and March 2022) enrolled pediatric patients with solid tumors or NHL treated with high‐intensity chemotherapy and with grade ≥3 myelosuppression for at least 14 days during chemotherapy. Prophylactic PEG‐rhG‐CSF was given at 100 μg/kg body weight (maximum total dosage of 6 mg) once 24–48 h following chemotherapy for two cycles. The primary endpoint was the incidence of PEG‐rhG‐CSF‐related adverse events (AEs). The key secondary endpoints were the rates of grade 3/4 neutropenia and febrile neutropenia (FN).

**Results:**

This study included 160 pediatric patients with a median age of 6.22 (0.29, 18.00) years. Fifty‐eight patients (36.25%) were diagnosed with sarcoma. AEs potentially related to PEG‐rhG‐CSF included bone pain (*n* = 32), fatigue (*n* = 21), pain at the injection site (*n* = 21), and myalgia (*n* = 20). The rates of grade 3/4 neutropenia and FN during treatment were 57.28% and 29.45%, respectively.

**Conclusion:**

PEG‐rhG‐CSF is well tolerated and effective in pediatric patients with solid tumors or NHL. These findings should be substantiated with further trials.

**Clinical Trial Registration:**

ClinicalTrials.gov identifier: NCT04547829.

## Graphical abstract

PEG‐rhG‐CSF was used to manage the chemotherapy‐induced neutropenia. The use of PEG‐rhG‐CSF (Jinyouli) was limited to Chinese adults. PEG‐rhG‐CSF as primary prophylaxis was tolerated well in pediatric patients.

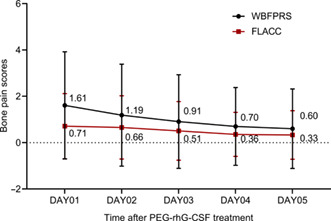

## 1 INTRODUCTION

Myelosuppression is a common potentially life‐threatening adverse effect (AE) of chemotherapy in childhood cancers.^1^ Chemotherapy‐induced neutropenia (CIN) is dose‐dependent and occurs in 26%–45% of treated individuals.^2^, ^3^ CIN is associated with high morbidity and mortality in children.^3^ Moreover, CIN can lead to chemotherapy interruption and dose adjustments, affecting the treatment outcomes.^4^

Recombinant human granulocyte colony‐stimulating factor (rhG‐CSF) is commonly used for reversing CIN,^5^, ^6^ but rhG‐CSF requires daily injections, affecting the compliance of pediatric patients. Polyethylene glycol conjugation (PEGylation) involves the covalent attachment of a 20‐kDa PEG moiety to the N‐terminal methionine residue of rhG‐CSF to prolong its half‐life and activity.^7^ Therefore, the plasma half‐life of PEG‐rhG‐CSF could be extended to up to 47 h, with preserved solubility, bioavailability, and stability.^8^ Previous studies demonstrated its cost‐effectiveness, safety, and comparable efficacy to conventional rhG‐CSF for the management of CIN in adults,^9^ children, and adolescents.^10^, ^11^

Pegfilgrastim, a PEGylated form of rhG‐CSF (analog of filgrastim), was approved for use in children to reverse CIN and reduce the incidence of neutropenia‐associated infections.^12^, ^13^ Although PEG‐rhG‐CSF (Brand name, Jinyouli) was approved for marketing in China in March 2012, its use has been limited to Chinese adults. In addition, the safety and efficacy of PEG‐rhG‐CSF (Jinyouli) in Chinese children receiving chemotherapy have not been broadly evaluated. Nevertheless, a previous study assessed the pharmacokinetics and pharmacodynamics of PEG‐rhG‐CSF (Jinyouli) in pediatric patients with acute lymphoblastic leukemia, providing some evidence supporting this treatment strategy in clinical practice.^14^

Therefore, this prospective multicenter phase II study aimed to examine the safety and efficacy of PEG‐rhG‐CSF as a primary prophylactic drug for neutropenia in pediatric patients with solid tumors or non‐Hodgkin lymphoma (NHL).

## 2 METHODS

### 2.1 Study design and patients

This prospective study enrolled pediatric cancer patients receiving high‐intensity chemotherapy resulting in grade ≥3 myelosuppression (determined by the investigators) in five clinical centers between October 2020 and March 2022.

The inclusion criteria were (1) ≤18 years of age, (2) confirmed cancer by histopathology or bone marrow cytology, (3) Eastern Cooperative Oncology Group (ECOG) score ≤2, (4) expected survival >8 months, (5) normal bone marrow hematopoietic function [absolute neutrophil count (ANC) ≥1.5 × 10^9^/L, platelet count (PLT) ≥80 × 10^9^/L, hemoglobin (Hb) ≥75 g/L, and white blood cell count (WBC) ≥3.0 × 10^9^/L], and (6) expected grade ≥3 myelosuppression after chemotherapy. The exclusion criteria were (1) local or systemic infection not adequately controlled, (2) severe visceral organ dysfunction, (3) allergy to PEG‐rhG‐CSF, rhG‐CSF, or other preparations or proteins expressed by *Escherichia coli*, (4) altered hematopoietic function after treatment with other drugs, (5) severe mental illness that might affect informed consent provision and/or adverse reaction observation, or (6) unsuitability to participate in this study as judged by the investigators.

This study was approved by the Ethics Committee of the Cancer Center of Sun Yat‐sen University (approval number: B2020‐202‐01). Informed consent was provided by each participant or their legal guardian. This trial was registered (ClinicalTrials.gov NCT04547829).

### 2.2 Treatment

Two cycles of chemotherapy were administered as the primary cancer treatment, and PEG‐rhG‐CSF [CSPC Baike (Shandong) Biopharmaceutical Co., Ltd., Shandong, China] was administered once 24–48 h after each cycle of chemotherapy as a prophylactic drug against neutropenia. The dosage was 100 μg/kg, not exceeding 6 mg. After total WBC recovered to >2 × 10^9^/L, neutrophil count to >0.8 × 10^9^/L, and platelet count to >80 × 10^9^/L, the subsequent course of chemotherapy was resumed. In case these values were not reached, a period of 7 days was allowed for the parameters to return to normal. If the above conditions were still not reached during the 7‐day period, the participants were withdrawn from the study.

### 2.3 Data collection and follow‐up

Blood routine and biochemical examinations, routine urinalysis, 15‐lead electrocardiography, and ECOG performance status score assessments were carried out 1 day before chemotherapy initiation. Subsequently, a routine blood examination was performed every other day following PEG‐rhG‐CSF treatment in each cycle until ANC was >0.5 × 10^9^/L. Delays in chemotherapy and dose adjustments were recorded.

The times from start to end for AEs as well as their severity, were also recorded. All participants underwent a safety assessment at the end of treatment.

### 2.4 Endpoints

The primary study endpoint was the incidence and severity of drug‐related AEs. Bone pain was scored daily by two scoring methods.^15^, ^16^, ^17^ In participants aged 8 years and over, the Wong–Baker's facial pain rating scale (WBFPRS) was applied for pain assessment; in those below 8, the Face, Legs, Activity, Cry, and Consolability (FLACC) scale was used.

The secondary endpoints were the incidence rate of grade 3/4 neutropenia in each chemotherapy cycle, ANC changes in each chemotherapy cycle, occurrence, and duration of febrile neutropenia (FN) in each chemotherapy cycle (axillary temperature was utilized as standard body temperature, and FN was defined as ANC <0.5 × 10^9^/L and axillary temperature >38°C), the lowest neutrophil count in each chemotherapy cycle, the incidence of chemotherapy delays or dose adjustments in each chemotherapy cycle, the proportion of participants administered antibiotics in all chemotherapy cycles, and the incidence rates and severity of AEs.

The AEs were assessed according to the National Cancer Institute (NCI) Common Terminology Criteria for Adverse Events (CTCAE) version 5.0. AEs occurring after treatment with the study drug, including symptoms, disease course, and laboratory abnormalities, were reported. The correlation between the study drug and AE occurrence was evaluated by the physicians according to a 5‐point scale.

In this study, subgroup analysis was performed to examine the differences in the incidence rates of grade 3/4 neutropenia and FN for different tumor types.

### 2.5 Statistical analysis

The full analysis set (FAS) included all participants who received the study drug at least once, with at least one efficacy evaluation available. The safety set (SS) included participants who received the study drug at least once, with safety assessments available after receiving the study drug.

Statistical analysis was performed with SPSS 19.0 (IBM, Armonk, NY, USA). Continuous data were presented as means ± standard deviations or medians (minimum, maximum) according to the distribution (normal or not). Categorical data were presented as *n* (%).

## 3 RESULTS

### 3.1 Patient clinicodemographic characteristics

**FIGURE 1:**
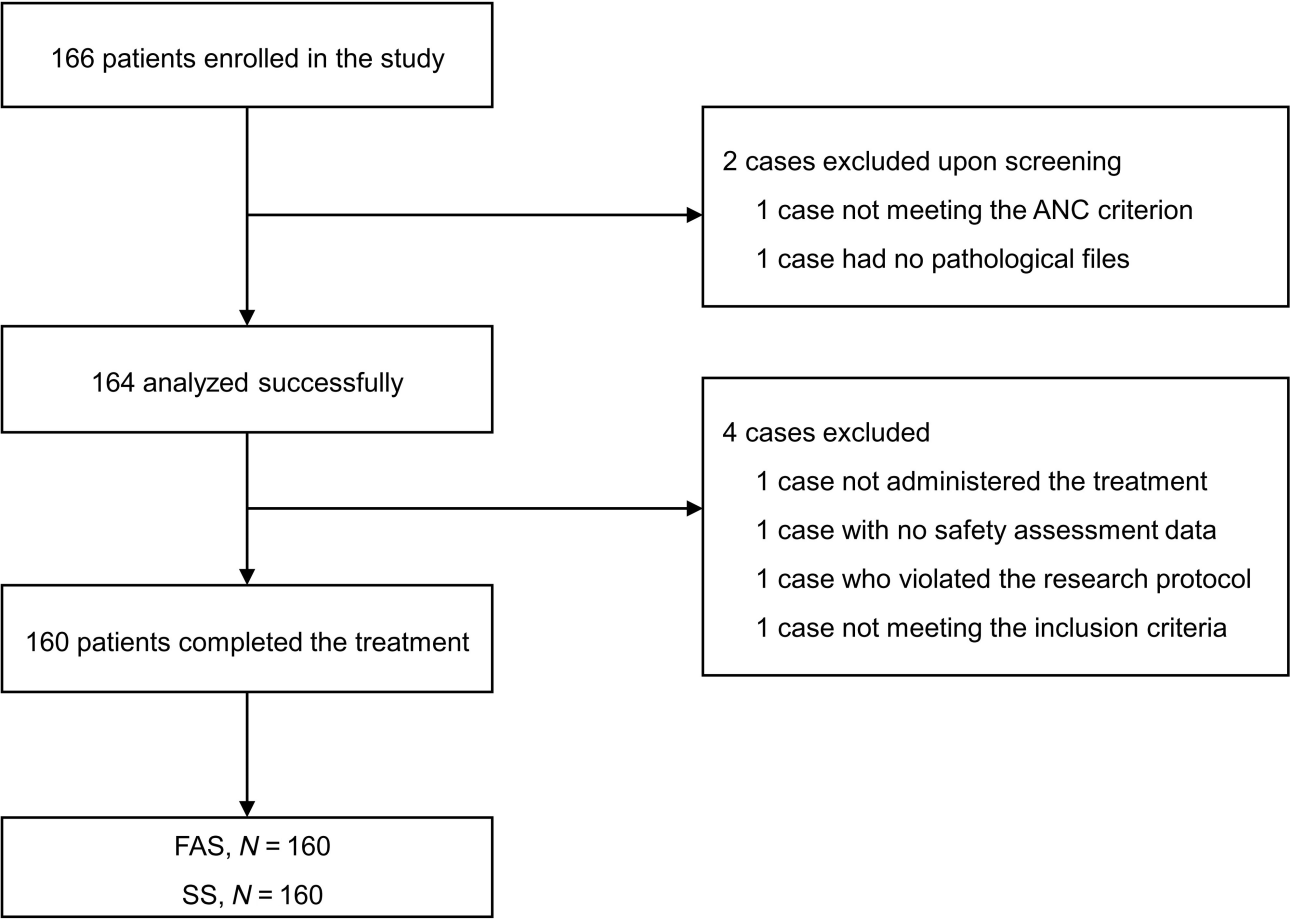
Study flowchart.

This study enrolled 166 participants, of whom two did not meet the inclusion criteria, and four did not complete the study. Eventually, 160 participants who completed the study treatment were included in the FAS and SS analyses (Figure 1).

The baseline characteristics of the included participants are shown in Table 1. The median age of the 160 participants was 6.22 (0.29, 18.00) years; 135 (84.38%) participants had an ECOG score of 1. Eighty‐seven (54.38%) participants had metastases, including 44 (27.50%) with metastases involving more than two organs. Thirty‐one (19.38%) participants received the cyclophosphamide + epirubicin + vincristine/ifosfamide + etoposide (CAV/IE) chemotherapy regimen.

**TABLE 1:** Baseline characteristics of the patients.

	*N* = 160
Age (years), Median (Min, Max)	6.22 (0.29, 18.00)
Sex	
Male, *n* (%)	90 (56.25)
Female, *n* (%)	70 (43.75)
ECOG score, *n* (%)	
0	8 (5.00)
1	135 (84.38)
2	11 (6.88)
Missing	6 (3.75)
Tumor type, *n* (%)	
Sarcoma	58 (36.25)
Neuroblastoma	33 (20.62)
Non‐Hodgkin Lymphoma	21 (13.12)
Germ cell tumor	19 (11.88)
Brain tumor	15 (9.38)
Hepatoblastoma and others	14 (8.75)
Metastases, *n* (%)	87 (54.38)
Number of organs with metastases, *n* (%)	
None	73 (45.62)
≤2	43 (26.88)
>2	44 (27.50)
Underlying diseases or comorbidities^a^, *n* (%)	16 (10.00)
History of drug allergy^b^, *n* (%)	8 (5.00)
Chemotherapy regimen, *n* (%)	
CAV/IE	31 (19.38)
CAV/VIP	21 (13.12)
CAV/CE	19 (11.88)
SCCCG‐BL	12 (7.50)
JEB	11 (6.88)
VAC/VII	9 (5.62)

Abbreviations: CAV/CE, cyclophosphamide + epirubicin + vincristine/ etoposide+ carboplatin; CAV/IE, cyclophosphamide + epirubicin + vincristine/ifosfamide + etoposide; CAV/VIP, cyclophosphamide + epirubicin + vincristine/ifosfamide + etoposide + cisplatin; JEB, etoposide + carboplatin + bleomycin; SCCCG‐BL, a modified BFM protocol for non‐Hodgkin lymphoma; VAC/VII, cyclophosphamide + actinomycin‐D + vincristine/ifosfamide + irinotecan (detailed chemotherapy regimens are available in supplementary materials).

^a^ Underlying diseases or comorbidities included thalassemia, hydrocele, nephrotic syndrome, chickenpox, and FAVA disease.

^b^ Allergy drugs included doxorubicin hydrochloride liposome injection, cephalosporin, amikacin, tetanus antitoxin, diphenhydramine, and albendazole.

### 3.2 Safety

All the AEs recorded during the study are detailed in Table 2. The most frequent drug‐related AE was bone pain (only grade 1–2), detected in 32 (20.0%) participants. Fatigue and pain at the injection site were observed in 21 (13.13%) participants each, and myalgia was observed in 20 (12.50%) participants. Other drug‐related AEs had rates below 10%, including fever (8.75%), dizziness (8.13%), joint pain (7.50%), and vomiting (5.00%) (Table S1). Grade 3 or 4 neutropenia were reported during the first cycle (71.88%), and the rate decreased to 41.61% in the second cycle (Table S2).

**TABLE 2:** Adverse events during the study (>10%).

	*N* = 160				
	G1	G2	G3	G4	Total
Anemia	15 (9.38)	73 (45.63)	60 (37.50)	0	148 (92.50)
Neutropenia	6 (3.75)	7 (4.38)	13 (8.13)	114 (71.25)	140 (87.50)
WBC count decrease	7 (4.38)	15 (9.38)	22 (13.75)	90 (56.25)	134 (83.75)
Lymphopenia	70 (43.75)	14 (8.75)	30 (18.75)	15 (9.38)	129 (80.63)
PLT count decrease	25 (15.63)	21 (13.13)	21 (13.13)	39 (24.38)	106 (66.25)
Febrile neutropenia	0	0	72 (45.00)	6 (3.75)	78 (48.75)
Lymphocytosis	49 (30.63)	4 (2.50)	0	0	53 (33.13)
Hyperphosphatemia	46 (28.75)	0	0	0	46 (28.75)
Fever	34 (21.25)	2 (1.25)	3 (1.88)	0	39 (24.38)
Bone pain	29 (18.13)	3 (1.88)	0	0	32 (20.00)
Fatigue	26 (16.25)	0 (0.00)	0	0	26 (16.25)
Myalgia	19 (11.88)	3 (1.88)	0	0	22 (13.75)
Pain at the injection site	20 (12.50)	1 (0.63)	0	0	21 (13.13)
Vomiting	19 (11.88)	1 (0.63)	0	0	20 (12.50)
Dizziness	20 (12.50)	0	0	0	20 (12.50)
Stomachache	17 (10.63)	1 (0.63)	0	0	18 (11.25)

**FIGURE 2:**
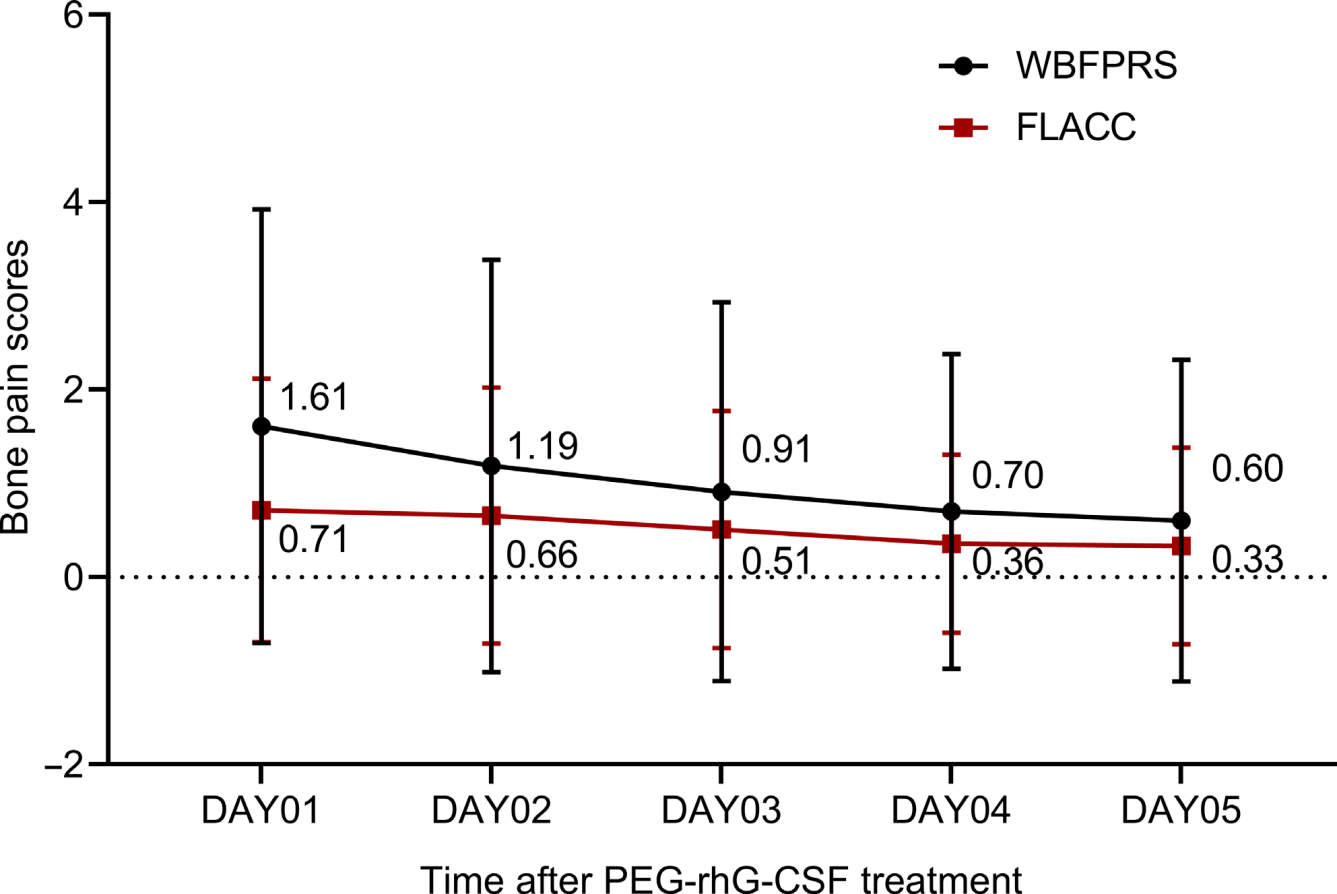
Bone pain scores.

Pain intensity was the highest the day after injection and decreased by 2.15‐fold (FLACC score, 0.71 vs. 0.33; *n* = 177) or 2.68‐fold (WBFPRS score, 1.61 vs. 0.60; *n* = 126) on day 5 (Figure 2). A similar trend in bone pain decrease was found in the days after injection in the different age groups.

### 3.3 Efficacy analysis

ANC peaked at 1 and 11 days after PEG‐rhG‐CSF treatment. ANC recovery occurred on average at 5.00 (1.00, 19.00) days in the first cycle and 4.00 (1.00, 14.00) in the second cycle (Table S3). Grade 3/4 neutropenia was observed in each chemotherapy cycle (Table S2).

Overall, the incidence of FN in all cycles was 29.45%. Seventy‐two (45.00%) patients had FN in the first cycle, and this number was reduced in the second cycle (19/149, 12.75%) (Table S2), for median durations of 3 and 2 days in the first and second cycles, respectively (Table S4). The incidence rates of grade 3/4 neutropenia and FN for different tumor types are shown in Tables S5 and S6. Patients with sarcoma and neuroblastoma had the highest incidence rates of grade 3/4 neutropenia and FN.

Chemotherapy delays occurred in two participants; treatment delay was due to an unrelated condition (bed shortage) in one patient, and the second case had hydrocephalus and required cerebrovascular outflow treatment and nutritional supplementation. No dose adjustment was needed.

Sixty‐six(41.25%) participants received antibiotics during myelosuppression in a total of 77 cycles, accounting for 24.92% (77/309) of all cycles. The participants were administered antibiotics for a therapeutic purpose (Table S7).

## 4 DISCUSSION

Pegfilgrastim is a PEG‐rhG‐CSF approved in the United States, but the documented evidence was based on very small sample size.^18^, ^19^ Only a few studies have reported its safety in Chinese patients.^14^, ^18^ In this study, PEG‐rhG‐CSF (Jinyouli) was given to 160 pediatric patients and administered high‐intensity chemotherapy as primary prophylaxis. A low overall incidence of drug‐related AEs was determined. To the best of our knowledge, this is the first prospective study of PEG‐rhG‐CSF performed in a large sample of Chinese pediatric patients with various solid tumors and lymphoma and showing promising outcomes after administration.

In previous studies, the main adverse reactions after PEG‐rhG‐CSF administration in adult and pediatric patients included bone pain, myalgia, fever, and fatigue.^20^, ^21^, ^22^, ^23^, ^24^, ^25^ In this study, no new safety signals were found, and AEs were comparable to those described in previous studies. The occurrence of bone and/or muscle pain is most likely attributed to the primary pharmacological effect of rhG‐CSF itself on the bone marrow^26^ and is likely not associated with PEGylation.^27^ The incidence of bone pain was slightly higher in the present study compared with a previous report,^20^ in which four out of 28 treated pediatric patients (median age, 14.5 years; range, 12–18 years) had bone pain. The participants in the present study were relatively younger (6.22 years, ranging from 0.29 to 18.00 years; 68 patients <5 years of age). In a previous large phase II study,^28^ 13 of the 38 enrolled patients were younger than 5 years. The present study further validates the safety and efficacy of PEG‐rhG‐CSF (Jinyouli) in young children. The slightly higher incidence of bone pain in this study than in previous reports may also be attributed to bone marrow development in young children.

The incidence and duration of severe neutropenia with PEG‐rhG‐CSF (Jinyouli) were comparable to pegfilgrastim in pediatric patients with sarcoma^29^ or after autologous peripheral blood stem cell transplantation.^30^ In a total of 218 cycles in the present study, the rate of FN following PEG‐rhG‐CSF (Jinyouli) administration (29.45%) was similar to that of children who received PEG‐rhG‐CSF (21.4%) for a total of 196 cycles.^19^ While no dose adjustments were performed due to neutropenia, chemotherapy was delayed in only two cases (2.1%), neither of which was associated with FN, which was fewer than in a previous study (9.4%).^20^ Drug efficacy differed in the present study by tumor type. Most patients in this study had sarcoma (*n* = 58), and FN in all cycles was found in 34 (35.51%) individuals, which was much less than reported for 25/37 patients (68%) with pegfilgrastim‐treated pediatric sarcoma after chemotherapy.^28^

Bone marrow suppression after high‐intensity chemotherapy for childhood tumors is the most common complication. PEG‐rhG‐CSF has the following advantages over rhG‐CSF. First of all, it reduces the number of injections, thereby reducing pain in pediatric patients and improving compliance. Secondly, compared with rhG‐CSF, PEG‐rhG‐CSF has a lower neutropenia rate in the subsequent chemotherapy cycle.^31^ Finally, the cost‐effectiveness of PEG‐rhG‐CSF for primary prevention is better than for secondary prevention because of the advantage of lowering FN incidence.^32^

This study has limitations. This study included patients with different tumor types and high‐intensity chemotherapy regimens. The sample size is too small, and the variability in cancers and chemotherapy regimens is too wide to allow any meaningful subgroup analysis.

In conclusion, prophylactic PEG‐rhG‐CSF can be used in pediatric cancer patients. The present study suggests that PEG‐rhG‐CSF injection is convenient, safe, and effective in pediatric patients with cancer receiving high‐intensity chemotherapies. Future studies are warranted to explore the efficacy differences among various tumor types.

## AUTHOR CONTRIBUTIONS

**Junting Huang:** Writing – original draft (lead); writing – review and editing (equal). **Suying Lu:** Data curation (equal); formal analysis (equal). **Juan Wang:** Data curation (equal); formal analysis (equal). **Liang Jiang:** Resources (equal); supervision (equal). **Xuequn Luo:** Resources (equal); supervision (equal). **Xiangling He:** Resources (equal); supervision (equal). **Yanpeng Wu:** Resources (equal); supervision (equal). **Yi Wang:** Investigation (equal); methodology (equal); software (equal); validation (equal). **Xiuli Zhu:** Data curation (equal); formal analysis (equal). **Jian Chen:** Data curation (equal); formal analysis (equal). **Yanlai Tang:** Data curation (equal); formal analysis (equal). **Keke Chen:** Data curation (equal); formal analysis (equal). **Xin Tian:** Data curation (equal); formal analysis (equal). **Boyun Shi:** Data curation (equal); formal analysis (equal). **Lanying Guo:** Data curation (equal); formal analysis (equal). **Jia Zhu:** Conceptualization (supporting); investigation (supporting). **Feifei Sun:** Conceptualization (supporting); investigation (supporting); methodology (supporting); writing – review and editing (supporting). **Zi‐jun Zhen:** Conceptualization (equal); investigation (equal); methodology (equal). **Yizhuo Zhang:** Conceptualization (lead); project administration (lead).

## FUNDING INFORMATION

The study was supported by CSPC Baike (Shandong) Biopharmaceutical Co., Ltd.

## CONFLICT OF INTEREST STATEMENT

The authors declare no conflict of interest.

## Supporting information


Table S1.
Table S2.
Table S3.
Table S4.
Table S5.
Table S6.
Table S7.

## Data Availability

All original data were deposited online http://www.researchdata.org.cn.

## References

[1] Phillips B . Prospective cohort study of the predictive value of inflammatory biomarkers over clinical variables in children and young people with cancer presenting with fever and neutropenia. F1000Res. 2021;10:1070. doi:10.12688/f1000research.73075.2 35211295 PMC8831847

[2] Sanguanboonyaphong P , Komvilaisak P , Suwannaying K , et al. Predictors of chemotherapy induced adverse events in pediatric osteosarcoma patients. Asian Pac J Cancer Prev. 2022;23(1):93‐100. doi:10.31557/APJCP.2022.23.1.93 35092376 PMC9258668

[3] Epstein RS , Weerasinghe RK , Parrish AS , Krenitsky J , Sanborn RE , Salimi T . Real‐world burden of chemotherapy‐induced myelosuppression in patients with small cell lung cancer: a retrospective analysis of electronic medical data from community cancer care providers. J Med Econ. 2022;25(1):108‐118. doi:10.1080/13696998.2021.2020570 34927520

[4] Bukowska‐Strakova K , Wlodek J , Pitera E , et al. Role of HMOX1 promoter genetic variants in Chemoresistance and chemotherapy induced neutropenia in children with acute lymphoblastic leukemia. Int J Mol Sci. 2021;22(3):988. doi:10.3390/ijms22030988 33498175 PMC7863945

[5] Moore L , Bartels T , Persky DO , Abraham I , Kumar A , McBride A . Outcomes of primary and secondary prophylaxis of chemotherapy‐induced and febrile neutropenia in bendamustine plus rituximab regimens in patients with lymphoma and chronic lymphocytic leukemia: real‐world, single‐center experience. Support Care Cancer. 2021;29(8):4867‐4874. doi:10.1007/s00520-020-05982-0 33547525

[6] N'Guessan‐Irie AG , Allah‐Kouadio E , Gnepehi OB , Kohi DS , Allouka KCE , Siransy‐Kouakou NG . Pharmacological management of adverse events during treatment of chronic viral hepatitis in three Ivorian University Hospitals. Int J Clin Pharmacol Ther. 2020;58(5):268‐273. doi:10.5414/CP203643 32101521

[7] Arvedson T , O'Kelly J , Yang BB . Design rationale and development approach for Pegfilgrastim as a long‐acting granulocyte colony‐stimulating factor. BioDrugs. 2015;29(3):185‐198. doi:10.1007/s40259-015-0127-4 25998211 PMC4488452

[8] Yang WY , Liu TF , Chen XJ , et al. Pharmacokinetics and pharmacodynamics of pegylated recombinant human granulocyte colony‐stimulating factor in children with acute lymphoblastic leukemia: a prospective control trial. Zhongguo Dang Dai Er Ke Za Zhi. 2020;22(11):1172‐1177. doi:10.7499/j.issn.1008-8830.2005048 33172550 PMC7666389

[9] Zhao J , Qiao G , Liang Y , et al. Cost‐effectiveness analysis of PEG‐rhG‐CSF as primary prophylaxis to chemotherapy‐induced neutropenia in women with breast cancer in China: results based on real‐world data. Front Pharmacol. 2021;12:754366. doi:10.3389/fphar.2021.754366 35185534 PMC8850939

[10] Thakkar D , Tiwari AK , Pabbi S , et al. Peripheral blood stem cell mobilization with pegylated granulocyte colony stimulating factor in children. Cancer Rep (Hoboken). 2021;4(6):e1408. doi:10.1002/cnr2.1408 34245131 PMC8714533

[11] Cho HW , Lee JW , Ju HY , et al. Safety and efficacy of Pegteograstim on chemotherapy‐induced neutropenia in children and adolescents with solid tumors. J Pediatr Hematol Oncol. 2022;44(2):e362‐e367. doi:10.1097/MPH.0000000000002206 34010932

[12] Fioredda F , Lanza T , Gallicola F , et al. Long‐term use of pegfilgrastim in children with severe congenital neutropenia: clinical and pharmacokinetic data. Blood. 2016;128(17):2178‐2181. doi:10.1182/blood-2016-07-727891 27621310

[13] Russell HV , Chi YY , Okcu MF , et al. Rising drug cost impacts on cost‐effectiveness of 2 chemotherapy regimens for intermediate‐risk rhabdomyosarcoma: a report from the Children's oncology group. Cancer. 2022;128(2):317‐325. doi:10.1002/cncr.33917 34623638 PMC8738099

[14] Yang WY , Liu TF , Chen XJ , et al. A single‐center, open‐label clinical study to evaluate pharmacokinetics and pharmacodynamics of pegylated recombinant human granulocyte stimulating factor in pediatric patients with acute lymphoblastic leukemia. J Clin Oncol. 2020;38(15_suppl):e22501. doi:10.1200/JCO.2020.38.15_suppl.e22501

[15] Nilsson S , Finnstrom B , Kokinsky E . The FLACC behavioral scale for procedural pain assessment in children aged 5‐16 years. Paediatr Anaesth. 2008;18(8):767‐774. doi:10.1111/j.1460-9592.2008.02655.x 18613934

[16] Merkel S , Voepel‐Lewis T , Malviya S . Pain assessment in infants and young children: the FLACC scale. Am J Nurs. 2002;102(10):55‐58. doi:10.1097/00000446-200210000-00024 12394307

[17] Wong DL , Baker CM . Pain in children: comparison of assessment scales. Okla Nurse. 1988;33(1):8. 3368206

[18] Liu XT , Zhao YX , Jia GW , et al. Pharmacokinetics and safety of pegylated recombinant human granulocyte colony‐stimulating factor in children with acute leukaemia. Br J Clin Pharmacol. 2021;87(8):3292‐3300. doi:10.1111/bcp.14750 33506975

[19] Schlenker L , Manworren RCB . Timing of Pegfilgrastim: association with febrile neutropenia in a pediatric solid and CNS tumor population. J Pediatr Oncol Nurs. 2021;38(6):375‐384. doi:10.1177/10434542211037729 34402328

[20] Andre N , Kababri ME , Bertrand P , et al. Safety and efficacy of pegfilgrastim in children with cancer receiving myelosuppressive chemotherapy. Anti‐Cancer Drugs. 2007;18(3):277‐281. doi:10.1097/CAD.0b013e328011a532 17264759

[21] Zou D , Guo M , Zhou Q . A clinical study of pegylated recombinant human granulocyte colony stimulating factor (PEG‐rhG‐CSF) in preventing neutropenia during concurrent chemoradiotherapy of cervical cancer. BMC Cancer. 2021;21(1):661. doi:10.1186/s12885-021-08364-9 34078317 PMC8173964

[22] Ma X , Kang J , Li Y , Zhang X . Pegfilgrastim safety and efficacy on the last chemotherapy day versus the next: systematic review and meta‐analysis. BMJ Support Palliat Care. 2021;1‐7. doi:10.1136/bmjspcare-2020-002532 34045224

[23] Park KH , Lee S , Park JH , et al. A randomized, multi‐center, open‐label, phase III study of once‐per‐cycle DA‐3031, a pegylated G‐CSF, in comparison with daily filgrastim in patients receiving TAC chemotherapy for breast cancer. Support Care Cancer. 2017;25(2):505‐511. doi:10.1007/s00520-016-3429-2 27709313

[24] Gu X , Zhang Y . Clinical efficacy and safety of mecapegfilgrastim in small cell lung cancer as primary prophylaxis of neutropenia post chemotherapy: a retrospective analysis. Ann Palliat Med. 2021;10(7):7841‐7846. doi:10.21037/apm-21-1400 34353071

[25] Ji X , Xu L , Pan P , Xu Z , Wang A , Li Y . Efficacy and safety of 3 mg pegylated recombinant human granulocyte colony‐stimulating factor as support to chemotherapy for lung cancer. Thorac Cancer. 2022;13(1):117‐125. doi:10.1111/1759-7714.14233 34791805 PMC8720626

[26] Nakov R , Gattu S , Wang J , Velinova M , Schaffar G , Skerjanec A . Proposed biosimilar pegfilgrastim shows similarity in pharmacokinetics and pharmacodynamics to reference pegfilgrastim in healthy subjects. Br J Clin Pharmacol. 2018;84(12):2790‐2801. doi:10.1111/bcp.13731 30079636 PMC6256001

[27] Li X , Zheng H , Yu MC , et al. Is PEGylated G‐CSF superior to G‐CSF in patients with breast cancer receiving chemotherapy? A systematic review and meta‐analysis. Support Care Cancer. 2020;28(11):5085‐5097. doi:10.1007/s00520-020-05603-w 32621264 PMC7333975

[28] Spunt SL , Irving H , Frost J , et al. Phase II, randomized, open‐label study of pegfilgrastim‐supported VDC/IE chemotherapy in pediatric sarcoma patients. J Clin Oncol. 2010;28(8):1329‐1336. doi:10.1200/JCO.2009.24.8872 20142595 PMC2834494

[29] Fox E , Widemann BC , Hawkins DS , et al. Randomized trial and pharmacokinetic study of pegfilgrastim versus filgrastim after dose‐intensive chemotherapy in young adults and children with sarcomas. Clin Cancer Res. 2009;15(23):7361‐7367. doi:10.1158/1078-0432.CCR-09-0761 19920107 PMC2787766

[30] Cesaro S , Nesi F , Tridello G , et al. A randomized, non‐inferiority study comparing efficacy and safety of a single dose of pegfilgrastim versus daily filgrastim in pediatric patients after autologous peripheral blood stem cell transplant. PLoS One. 2013;8(1):e53252. doi:10.1371/journal.pone.0053252 23308174 PMC3538773

[31] Xie J , Cao J , Wang JF , et al. Advantages with prophylactic PEG‐rhG‐CSF versus rhG‐CSF in breast cancer patients receiving multiple cycles of myelosuppressive chemotherapy: an open‐label, randomized, multicenter phase III study. Breast Cancer Res Treat. 2018;168(2):389‐399. doi:10.1007/s10549-017-4609-6 29230663

[32] Wu Q , Li Q , Zhang J , et al. Comparison of primary and secondary prophylaxis using PEGylated recombinant human granulocyte‐stimulating factor as a cost‐effective measure in malignant neoplasms: a multicenter retrospective study. Front Pharmacol. 2021;12:690874. doi:10.3389/fphar.2021.690874 34776940 PMC8586644

