# Supplementary material for: A multicenter phase II trial of primary prophylactic PEG‐rhG‐CSF in pediatric patients with solid tumors and non‐Hodgkin lymphoma after chemotherapy: An interim analysis
Source: Cancer Med. 2023 May 15;12(13):14130–7. doi: 10.1002/cam4.6079 (PMC10358250; doi:10.1002/cam4.6079)
Supplement: Supplementary file 1 — Table S1. Table S2. Table S3. Table S4. Table S5. Table S6. Table S7. [file CAM4-12-14130-s001.docx]

**Supplementary** **Table 1.** Incidence rates and severity of drug-related adverse events (AEs) in pediatric patients with cancer (n=160)

|  | AEs n (%) | | | | |
| --- | --- | --- | --- | --- | --- |
|  | G1 | G2 | G3 | G4 | Total |
| Bone pain | 29 (18.12) | 3 (1.88) | 0 | 0 | 32 (20.00) |
| Fatigue | 21 (13.13) | 0 | 0 | 0 | 21 (13.13) |
| Pain at the injection site | 20 (12.50) | 1 (0.63) | 0 | 0 | 21 (13.13) |
| Myalgia | 18 (11.25) | 2 (1.25) | 0 | 0 | 20 (12.50) |
| Fever | 12 (7.50) | 1 (0.63) | 1 (0.63) | 0 | 14 (8.75) |
| Dizziness | 13 (8.13) | 0 | 0 | 0 | 13 (8.13) |
| Joint pain | 10 (6.25) | 1 (0.63) | 1 (0.63) | 0 | 12 (7.50) |
| Vomiting | 8 (5.00) | 0 | 0 | 0 | 8 (5.00) |
| Stomachache | 3 (1.88) | 0 | 0 | 0 | 3 (1.88) |
| Toothache | 3 (1.88) | 0 | 0 | 0 | 3 (1.88) |
| Induration at the injection site | 3 (1.88) | 0 | 0 | 0 | 3 (1.88) |
| Facial pain | 2 (1.25) | 0 (0.00) | 0 | 0 | 2 (1.25) |
| Insomnia | 2 (1.25) | 0 (0.00) | 0 | 0 | 2 (1.25) |
| Nausea | 2 (1.25) | 0 | 0 | 0 | 2 (1.25) |
| Allergic rash | 1 (0.63) | 1 (0.63) | 0 | 0 | 2 (1.25) |
| Diarrhea | 1 (0.63) | 0 | 0 | 0 | 1 (0.63) |
| Muscle spasms | 1 (0.63) | 0 | 0 | 0 | 1 (0.63) |
| Headache | 1 (0.63) | 0 | 0 | 0 | 1 (0.63) |
| Limb pain | 1 (0.63) | 0 | 0 | 0 | 1 (0.63) |
| Leukocytosis | 0 (0.00) | 0 | 1 (0.63) | 0 | 1 (0.63) |
| Polypnea | 1 (0.63) | 0 | 0 | 0 | 1 (0.63) |
| Lymphocyte count decreased | 1 (0.63) | 0 | 0 | 0 | 1 (0.63) |

AEs, adverse events

**Supplementary Table 2.** Incidence rates of grade 3/4 neutropenia and febrile neutropenia

|  | N | Grade 3 or 4 neutropenia, n (%) | Febrile neutropenia, n (%) |
| --- | --- | --- | --- |
| Cycle 1 | 160 | 115 (71.88) | 72 (45.00) |
| Cycle 2 | 149 | 62 (41.61) | 19 (12.75) |
| All cycles | 309 | 177 (57.28) | 91 (29.45) |
| All patients | 160 | 127 (79.38) | 78 (48.75) |

**Supplementary Table 3. Number of days until** **the absolute neutrophil count (ANC) recovery**

|  | Cycles | ANC recovery time (days) |
| --- | --- | --- |
| Cycle 1 Median (Min, Max) | 129 | 5.00 (1.00, 19.00) |
| Cycle 2 Median (Min, Max) | 76 | 4.00 (1.00, 14.00) |
| Total cycles  Median (Min, Max) | 205 | 4.00 (1.00, 19.00) |
| Total number of patients Median (Min, Max) | 135 | 6.0 (1.00, 26.00) |

ANC, absolute neutrophil count

**Supplementary Table 4.** Duration of febrile neutropenia in each chemotherapy cycle

|  | FN duration (days) |
| --- | --- |
| Cycle 1 Median (Min, Max) | 3.00 (1.00, 12.00) |
| Cycle 2 Median (Min, Max) | 2.00 (1.00, 7.00) |
| Total cycles Median (Min, Max) | 2.00 (1.00, 12.00) |
| Total number of patients  Median (Min, Max) | 3.00 (1.00, 18.00) |

FN, febrile neutropenia

**Supplementary Table 5.** Incidence of Grade 3/4 neutropenia in different types of tumors

| Grade 3/4 neutropenia | Sarcoma  (N=58) | Neuroblastoma (N=33) | Lymphoma (N=21) | Germ cell tumor (N=19) | Brain tumor  (N=15) | Others  (N=14) |
| --- | --- | --- | --- | --- | --- | --- |
| Cycle 1 | 58 | 33 | 21 | 19 | 15 | 14 |
| Occurrence | 43 (74.14) | 29 (87.88) | 16 (76.19) | 12 (63.16) | 7 (46.67) | 8 (57.14) |
| Cycle 2 | 55 | 32 | 20 | 18 | 11 | 13 |
| Occurrence | 22 (40.00) | 18 (56.25) | 10 (50.00) | 1 (5.56) | 5 (45.45) | 6 (46.15) |
| Total cycles | 113 | 65 | 41 | 37 | 26 | 27 |
| Occurrence | 65 (57.52) | 47 (72.31) | 26 (63.41) | 13 (35.14) | 12 (46.15) | 14 (51.85) |
| Total number of patients | 58 | 33 | 21 | 19 | 15 | 14 |
| Occurrence | 47 (81.03) | 31 (93.94) | 16 (76.19) | 13 (68.42) | 10 (66.67) | 10 (71.43) |

**Supplementary Table 6.** Incidence of febrile neutropenia in different types of tumors

| FN | Sarcoma  (N=58) | Neuroblastoma  (N=33) | Lymphoma  (N=21) | Germ cell tumor  (N=19) | Brain tumor  (N=15) | Others  (N=14) |
| --- | --- | --- | --- | --- | --- | --- |
| Cycle 1 | 58 | 33 | 21 | 19 | 15 | 14 |
| Occurrence | 32 (55.17) | 18 (54.55) | 10 (47.62) | 5 (26.32) | 3 (20.00) | 4 (28.57) |
| Cycle 2 | 55 | 32 | 20 | 18 | 11 | 13 |
| Occurrence | 7 (12.739) | 6 (18.75) | 4 (20.00) | 0 (0.00) | 2 (18.18) | 0 (0.00) |
| Total cycles | 113 | 65 | 41 | 37 | 26 | 27 |
| Occurrence | 39 (34.51) | 24 (36.92) | 14 (34.15) | 5 (13.51) | 5 (19.23) | 4 (14.81) |
| Total number of patients | 58 | 33 | 21 | 19 | 15 | 14 |
| Occurrence | 34 (58.62) | 20 (60.61) | 10 (47.62) | 5 (26.32) | 5 (33.33) | 4 (28.57) |

FN, febrile neutropenia

**Supplementary Table 7.** The proportion of patients who received antibiotics

| Antibiotics usage | N (%) |
| --- | --- |
| Total cycles | 309 |
| Usage | 77 (24.92) |
| Total number of patients | 160 |
| Usage | 66 (41.25) |
